# Entrepreneurship education and entrepreneurial intention of Chinese college students: Evidence from a moderated multi-mediation model

**DOI:** 10.3389/fpsyg.2022.1049232

**Published:** 2022-11-29

**Authors:** Yuan Gao, Xiao Qin

**Affiliations:** ^1^Business School, Lingnan Normal University, Zhanjiang, China; ^2^Guangdong Coastal Economic Belt Development Research Center, Zhanjiang, China; ^3^Nanjing University of Science and Technology ZiJin College, Nanjing, China

**Keywords:** entrepreneurship education, entrepreneurial self-efficacy, entrepreneurial intention, entrepreneurship competition, family income, Chinese college students

## Abstract

Entrepreneurship plays an active role in promoting economic and population integration and social mobility. To further promote economic and social development, the Chinese government and universities have launched entrepreneurship education courses and encouraged college students to participate in entrepreneurship competitions to enhance their entrepreneurial knowledge, entrepreneurial ability and entrepreneurial intention. However, the entrepreneurial intention of Chinese college students is still not high. Therefore, a question arises: How should entrepreneurial education be carried out? Can entrepreneurial competitions and entrepreneurial self-efficacy be an effective medium in augmenting entrepreneurial education on entrepreneurial intention? Is family income an effective moderator affecting college students’ entrepreneurial intention? To answer these questions, this study used quantitative methods to collect 351 sample data points, and a theoretical model was constructed to explain the mechanism forming entrepreneurial education and entrepreneurial intention. The results show that entrepreneurial self-efficacy plays a partial mediating role between entrepreneurial education and entrepreneurial intention, entrepreneurial competition and entrepreneurial self-efficacy play a chain mediating role and family income positively moderates the relationship between entrepreneurial education and entrepreneurial intention. The contribution of this study is to reveal the black box of the formation mechanism in college students’ entrepreneurial intentions, affirms the role of the Chinese government in promoting entrepreneurial competitions and provides empirical evidence for the effective development of entrepreneurial practise activities, as well as theoretical references for entrepreneurial policy makers.

## Introduction

Entrepreneurship is a mechanism used to promote economic growth, cultural formation, population integration and social mobility (Reynolds et al., 2005), and it has drawn increasing attention from policy makers (Nowiński et al., 2019). Especially in the face of global economic pressure, entrepreneurship is an effective way to promote economic transformations through innovative achievements and relieve employment pressure. College students, as a new force in the future development of a country, should become the main force in creating jobs. Guiding and cultivating college students’ entrepreneurship has become an important policy orientation in China. However, according to the 2021 Chinese College Students Entrepreneurship Report, 96.1% of Chinese college students have had entrepreneurial ideas, but only 14% have actually implemented them. Most of their entrepreneurial motivations come from the potential high income, the possibility of fame, their desire to enjoy free time and their wish to escape part-time jobs. These motivations are not the positive internal driving forces required to foster entrepreneurship. Therefore, we believe that Chinese college students still lack a current internal driving force for entrepreneurship. This is also a problem relevant to the implementation of innovation and entrepreneurship. Some studies have shown that entrepreneurial intention (EI) as a behavioural intention has significant explanatory power for entrepreneurial activities (Liñán and Fayolle, 2015), whilst entrepreneurship education (EE) is regarded as an effective method to develop and encourage entrepreneurship (Boldureanu et al., 2020). The European Union (EU) sees innovation and entrepreneurship as lifelong learning mandates (European Commission, 2013). In China, to encourage college students to start their own businesses, EE has been integrated into college classrooms and has become a compulsory or elective course for college students, helping college students better plan their time and future career development (Ratten and Usmanij, 2021). The aim of these endeavours is to solve the lack of internal motivation amongst Chinese college students to encourage them to start a business, which fundamentally promotes college students’ entrepreneurship.

Entrepreneurial competitions (ECs) are seen as an important factor driving entrepreneurial decision-making by entrepreneurs (Urbig et al., 2019). These competitions require participants to work individually or in teams to develop a new entrepreneurial idea or a new business plan; then, following judgement by the jury (Watson and McGowan, 2019), students motivated by the EC will be more willing to participate in business activities and participate in entrepreneurship (Bergmann et al., 2018). In addition, some scholars believe that entrepreneurial self-efficacy (ESE) plays an important role in determining careers and whether to carry out entrepreneurial activities. ESE is different from general self-efficacy, as it influences career development and performance, or occupational self-efficacy (Newman et al., 2019).

Although there is relevant evidence that ESE and EC play important roles in the relationship between EE and EI, there are still some research gaps in the literature. On the one hand, there is little evidence pointing to the need for universities to launch ECs. Universities around the world have successively launched initiatives to support entrepreneurial projects, and German universities spend more than 7,500 euros per year on business plan competitions and related implementations and policies (Brooks et al., 2009; Frank et al., 2017). China also holds competitive ECs, such as the Innovation and Entrepreneurship Creativity e-commerce competition, Internet+ competition and Innovation and Entrepreneurship Competition (referred to as Da Chuang Competition). The value of ECs in traditional universities has been questioned (Watson and McGowan, 2019), so it is necessary to determine the mechanisms through which ECs influence EI. On the other hand, the moderating effect of family economic factors on college students’ EI has not been well studied. The existing literature rarely mentions the influence of family economic status on EI, but its impact on students’ EI is an issue that cannot be ignored. Therefore, based on the research of some scholars (e.g., Nowiński et al., 2019; Hoang et al., 2020; Li et al., 2022), this study introduces the variable of family income as a contextual factor that affects EE and EI to determine whether there are differences in EI in the case of different family incomes. The above are the research gaps to be filled in this paper, which aims to reveal the black box of the formation mechanism of EI amongst college students, enrich the relevant literature on the development of EI and entrepreneurial ability, provide empirical evidence for the effective development of entrepreneurial practise activities and provide theoretical references for entrepreneurial policy makers. In view of this, our research questions are as follows:

1. Are EC and ESE an effective medium in promoting EE and EI?
2. Does family income moderate the relationship between EE and EI?

To answer the above questions, we collected data based on scales for relevant indicators. Using these indicators, we designed survey items (Table 1) and conducted questionnaire collection amongst Chinese college students. The research results showed that (1) EE and EI play a mediating role, and the chain mediating effect of the two is greater than the single mediating effect of ESE; and (2) family income positively moderates the relationship between EE and EI, as well as ESE and EI.

**Table 1:** Variable factor loading, cumulative variance explained rate (after rotation) and source.

Variable	Indices	Factor load	Cumulative variance interpretation rate (after rotation)	Sources
EI	My goal is to be an entrepreneur Y1	0.800	20.981	Liñán and Chen (2009); Lu et al. (2013)
	I will try my best to start a business Y2	0.797		
	I’m ready to start a business Y3	0.765		
	Even if the startup fails, I will continue to work hard until I succeed Y4	0.618		
	Even if my parents oppose me, I will still devote myself to starting my own business Y5	0.695		
EE	Entrepreneurship course types have a variety of course types X1	0.687	16.037	Nichols and Armstrong (2003); Cheung (2008); Seikkula-Leino et al. (2015); Byun et al. (2018)
	Entrepreneurship courses use a variety of teaching methods X2	0.682		
	Teachers have rich experience in entrepreneurship teaching when teaching entrepreneurship courses X3	0.730		
	Entrepreneurship course content is closely integrated with majors X4	0.614		
	Entrepreneurship course content keeps up with the forefront of the times X5	0.709		
ESE	If I start a business, I will have a great chance of success M11	0.655	12.246	Liñán and Chen (2009); Official Journal of the European Union (OJEU) (2006); Lackeus (2015); Li et al. (2022)
	Past work experience helped me to start a business M12	0.640		
	I believe it is easier to start a business M13	0.61		
	I believe that I can choose an industry with potential to start a business M14	0.539		
EC	I would love to participate in entrepreneurship competitions M21	0.738	11.542	Hasan et al. (2017); Watson and McGowan (2017)
	I am very willing to participate in group entrepreneurship practise M22	0.798		
	Entrepreneurship competitions make it easier for my projects to land M23	0.555		
	Entrepreneurial competitions are highly integrated with majors M24	0.636		
Total explained variance: 60.806				

The common factors were extracted based on the principle of eigenvalues >1, whilst the factor loading matrix was rotated using the maximum variance method.

The rest of the paper is organised as follows: Section 2 explains the theoretical background and hypothesis development. Section 3 outlines the methodology. Section 4 presents the results of the data analysis. Section 5 is the discussion, which includes the implications, research limitations and directions for future research. Finally, Section 6 concludes the research.

## Theoretical background and development of hypotheses

### Perspectives of human capital theory and motivation theory

The theory of human capital was proposed by Theodore W. Schultz of the United States at the American Economic Association in 1960. Economists generally believe that human capital investments can promote economic growth (Becker, 1964), and the field of human capital does not only consider productivity and investment behaviour (McLeod and Nite, 2019). It also highlights how the sum of the opportunity costs of education, training and education act as a producer, and productivity in non-market situations is changed by education and investment in knowledge (Walter and Block, 2016). Many related studies have shown that there is a positive relationship between high-level knowledge, high-quality education and the labour market (Martin et al., 2013; Walter and Block, 2016). In the process of delivering EE to college students, fully understanding the theory of human capital will help to further elucidate the impact of EE on entrepreneurial behaviour.

Motivation theory was proposed by Woodworth in 1918. The theory believes that motivation is generated by individual needs, and when this need reaches a certain level, it is transformed into motivation. Scholars believe that motivation is the key to the formation of EI (Li and Zeng, 2018). For college students, their entrepreneurial motivation basically comes from internal spontaneous needs. The satisfaction brought about by EE includes the enrichment of entrepreneurial knowledge and theories. Furthermore, the sense of achievement that ECs provide through the acquisition of experience and skills can stimulate individuals’ need for self-growth and self-realisation. These factors help to form entrepreneurial motivation and convert it into EI.

### The relationship between EE, EC and EI

In human capital and economic growth theory, education and training are considered investments that increase efficiency and profitability. Schultz (1960) proposed that improving the quality and knowledge of the population is of great significance to well-being and that education is an important investment in improving the quality and knowledge of the population. Later, Becker (1964) affirmed this view. Education is a key factor in human capital due to its positive contributions to productivity improvements and human capital accumulation (North, 1990), but education is not limited to one model. Learning-by-doing models can also be used to accumulate human capital (Lucas, 1988). EE is usually delivered through the learning by doing model, which can effectively cultivate students’ creative skills and increase the possibility of future entrepreneurship. In the process of cognitive learning related to entrepreneurship, students participating in EE courses and entrepreneurship training provided by institutions can improve their entrepreneurial awareness and enrich their understanding of entrepreneurial activities. Li et al. (2018) proposed, based on research on college students’ entrepreneurial parks, that individuals transform past experience into entrepreneurial knowledge through practical learning, which is a process of exploration and trial and error, and that past experience can stimulate the entrepreneurial ideas of college students. Therefore, we can conclude that college students can increase the accumulation of knowledge and experience through entrepreneurial learning, improve students’ skills and abilities and help them understand their own entrepreneurial activities, which can then directly or indirectly affect their EI.

As the practical application of EI, EC is a way to simulate entrepreneurial behaviour, which can develop students’ creative thinking and entrepreneurial abilities (Fretschner and Weber, 2013) whilst improving their teamwork skills (Li and Wu, 2019). It is an exercise to increase experience and promote future entrepreneurship. Academia has seen ECs as an element of the entrepreneurial ecosystem (Dif et al., 2018). Therefore, considering the influence of EC on EI, it may be a mediating variable between EE and EI. Based on this, we propose the following hypotheses:

*H1*: EE is positively related to EI (main effect).

*H2*: EE is positively related to ECs.

*H3*: ECs are positively related to EI. Hence, ECs mediate the relationship between EE and EI.

### Relationship between EE, ESE and EI

With the further development of human capital theory, researchers have found that cognitive ability can effectively improve workers’ productivity in completing standardised work tasks (Coyle et al., 2018). Furthermore, non-cognitive ability can effectively improve workers’ completion of non-standardised work tasks and the labour productivity of the entire work organisation (Yan, 2020). Non-cognitive ability has stronger plasticity than cognitive ability, and education is an important means of ability formation. Therefore, the impact of education on non-cognitive ability may be greater than that on cognitive ability (Yu et al., 2017). Saeed et al. (2015) believe that EE provides students with entrepreneurial awareness, motivation and conceptual development support for business ideas in the early stage of entrepreneurship, which is conducive to the formation of ESE. The higher the ESE of entrepreneurial individuals, the stronger their intention to start a business and the more confident they will be in the success of entrepreneurship (Xu and Hao, 2019). Therefore, ESE is one of the key factors affecting entrepreneurial behaviour and has an important impact on entrepreneurial orientation. In summary, EE has a certain positive effect on the formation of ESE, and ESE can promote the generation of entrepreneurial motivation to a large extent. Some scholars even believe that ESE has a complete intermediary effect between EE and EI (Wu et al., 2021). Therefore, we believe that ESE may also play a mediating role between EE and EI. In view of this, we propose the following hypothesis:

*H4*: EE is positively related to ESE.

*H5*: ESE is positively related to EI; hence, ESE mediates the relationship between EE and EI.

### Relationship between ECs and ESE

As mentioned earlier, ECs are crucial to developing students’ abilities. Research shows that environments outside the classroom are more conducive to fostering students’ creativity (Davies et al., 2013). In EC, students can identify their own shortcomings and gain opportunities to learn from others (Watson and McGowan, 2017). Sukiennik et al. (2021) studied raw material competitions held in Poland from 2019 to 2022 and found that training on projects and initiatives within relevant frameworks is beneficial in raising awareness amongst participants. Furthermore, research has shown that participating in business plan competitions has a significant positive effect on non-management students (Zhao et al., 2022). Students can also develop entrepreneurial skills by participating in customised ECs (Treanor et al., 2021). Researchers have determined that actively organising and cultivating students’ growth by encouraging their participation in various types of ECs is beneficial for increasing students’ entrepreneurial ability and entrepreneurial confidence. Thus, we propose the following hypothesis:

*H6*: EC is positively related to ESE.

### The moderating effect of family income on EE and EI

Population factors, including gender, being an only child, parental entrepreneurial experience, family income and other socioeconomic characteristics, are critical in enhancing EI or entrepreneurial behaviour (Širola, 2020; Akman, 2021). Research has shown that every year, more than 50% of potential entrepreneurs choose to give up their entrepreneurial ideals due to a lack of financial support (Farooq et al., 2018). For college students who are relatively lacking in social resources, their start-up funds basically come from family support or start-up loans (Guo et al., 2021). Therefore, poor family economic conditions may weaken the strength of family support. Based on this, we believe that family financial support has a positive moderating effect on college students’ EI:

*H7a*: Family income positively moderates the relationship between EE and EI.

*H7b*: Family income positively moderates the relationship between ESE and EI.

### Conceptual model

The main purpose of our study was to investigate the effect of EE implementation amongst Chinese college students and to explore the relationship between EE and EI. In addition to the mediating effect of EC and ESE, as mentioned earlier, and the moderating effect of family income, we also hypothesise a direct relationship between EE and EI:

*H8*: The relationship between EE and EI is mediated by EC and ESE.

**Figure 1:**
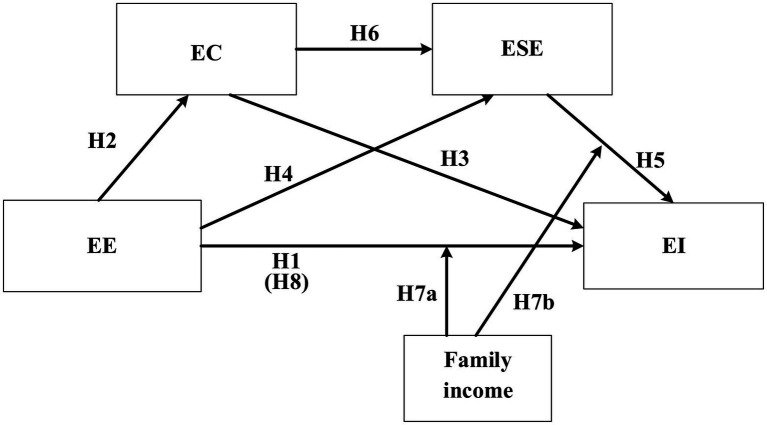
Hypothesised model.

We then developed a conceptual model that included all hypotheses (Figure 1).

## Materials and methods

### Sample and data collection

To test our hypotheses, we enlisted the assistance of a professional sample collection service platform called Wen Juanxing to help collect questionnaires. This platform is one of the largest questionnaire collection service platforms in China. Its customers include more than 30,000 enterprises and 90% of the universities in the country, and it provides a variety of online questionnaire services with a high reputation in China, which is the main reason we chose this platform. The questionnaire we chose used the cluster sampling technique; that is, the paid service Questionnaire Star was used to lock the age group of respondents to 18–22 years and their occupation to student. This guaranteed that our questionnaire respondents were college students, and the system then randomly chose respondents from a given pool. Users who met these two criteria were issued an online questionnaire. According to the statistics collected from the questionnaires, the questionnaires covered 34 provinces and municipalities across the country. Respondents belonged to various multi-disciplinary fields, such as science, engineering, business, literature and art, avoiding cultural differences caused by regions and disciplines. Our questionnaire yielded a total of 380 samples. According to the trap topic, the invalid questionnaire was removed, and the remaining number of valid questionnaires was 351. The efficiency of the questionnaire was 92.4%, which met the requirements for empirical research (Baruch, 1999).

### Variable measurement

The questionnaire covers four dimensions, EE, ESE, EI and EC. According to previous related research, 18 indicator variables were selected. The source of each indicator, the load factor of each variable and the cumulative variance explained rate (after rotation) are shown in Table 1.

### Sample description

The participants in the sample were college students, most of whom were from East China (23.08%) and Central China (18.23%), with the smallest group being from Northeast China (4.27%) and Northwest China (4.27%). Roughly 27.6% of respondents were only children. Having parents with entrepreneurial experience accounted for 45% of the group. Freshman students accounted for 8.55% of all survey respondents (given that the survey was sent in July, freshman students had already participated in EE courses). Sophomores and juniors were the main subjects of this survey, accounting for 30.2 and 34.47%, respectively. In terms of majors, engineering students accounted for the largest number (32.19%), followed by science (18.8%), economics and management (18.23%) and philosophy (0.28%). In terms of gender, more women (52.4%) participated than men (47.6%). In addition, 92.5% of students had received education in entrepreneurship courses, and 99.97% of students knew that there were ECs.

Descriptive statistics were carried out for the variables related to the four dimensions: EE, ESE, EI and EC. All indicators were judged using a 7-point Likert scale, with 1 being completely disagree and 7 being completely agree. The average value for EE was 4.98–5.38, which is higher than the average value for other dimensions, indicating that EE is popular in China, and the average value for ‘entrepreneurship course content closely following the frontier of the times’ was the highest, indicating the combination of EE courses that respond to corresponding modern developments. The students found the level to be very high and were quite satisfied. The mean value of ESE ranged from 4.3 to 5.19, indicating that students’ self-recognition of entrepreneurial ability was above average and that Chinese college students had relatively good ESE. The average value for ECs was 3.73–4.57, of which the score for ‘I am very willing to participate in group entrepreneurship competitions’ was the lowest, and the score for ‘I am very willing to participate in entrepreneurship competitions’ was the highest, indicating that students were more inclined to simulate entrepreneurship than engage in practical exercises. This may be due to other factors, such as academic pressure or time constraints. The average value for EI ranged from 3.36–5.32 and fluctuated the most in several dimensions, indicating that students’ EI is different. The score for ‘I will try my best to start a business’ was the lowest, and the score for ‘Even if my parents oppose, I will still devote myself to starting my own business’ was the highest, showing that when students choose to give up their entrepreneurship goals, parental opposition may not be the main factor for abandonment (Table 2).

**Table 2:** Summary of the sample’s characteristics.

Variable	Minimum	Maximum	Mean		Variance statistics
			Statistics	Standard error	
X1	1	7	5.32	0.058	1.191
X2	1	7	5.11	0.069	1.650
X3	1	7	5.08	0.072	1.814
X4	1	7	4.98	0.074	1.925
X5	1	7	5.38	0.071	1.762
X6	1	7	5.13	0.083	2.428
M11	1	7	5.19	0.078	2.157
M12	1	7	5.03	0.072	1.811
M13	1	7	4.43	0.075	1.995
M14	1	7	4.30	0.072	1.845
M21	1	7	4.57	0.085	2.560
M22	1	7	3.73	0.083	2.408
M23	1	7	4.52	0.079	2.199
M24	1	7	4.01	0.098	3.388
Y1	1	7	4.33	0.099	3.472
Y2	1	7	3.36	0.094	3.076
Y3	1	7	4.11	0.091	2.899
Y4	1	7	3.70	0.094	3.129
Y5	1	7	5.32	0.058	1.191

### Reliability and validity test

The reliability of the questionnaire was determined by calculating the Cronbach’s α value, convergent reliability and construct reliability of each dimension. Table 3 shows that the Cronbach’s α for each variable was higher than 0.7, and the CR value was close to or higher than 0.8, indicating that the scale had a good level of reliability. The average variance (AVE) was close to or higher than 0.5, combined with the factor loading and total variance explained rate, indicating that there was a certain correlation between variables and that each dimension and variable could be explained consistently. The Kaiser–Meyer–Olkin (KMO) values were all >0.7, which is suitable for further factor analysis.

**Table 3:** Reliability test index.

	Cronbach’s α	KMO	AVE	CR
EE	0.794	0.815	0.437	0.795
ESE	0.730	0.721	0.416	0.739
EC	0.765	0.712	0.484	0.782
EI	0.862	0.841	0.558	0.863

Since the data came from a questionnaire, we performed a common method bias test on the data and used Herman’s single-factor test to conduct principal factor analysis for the items involved. The explanation rate of the first principal factor was 38.5% (without rotation), less than 40% of the cut-off point suggested by Hair et al. (2019), indicating that there was no serious common method bias in this study. Next, to ensure the validity of the hypothesis testing, confirmatory factor analysis was used to judge model fit. The results were as follows: Chi-squared = 368.924, Df = 129, Chi-square/df = 2.860 (less than 3; Bagozzi and Yi, 1988; Bentler, 1990), CFI = 0.908 (>0.9), TLI = 0.891 (close to 0.9), SRMR = 0.055, and RMSEA = 0.073 (less than 0.08; Browne and Cudeck, 1992). Therefore, the results show that the model fits well.

The data from the questionnaire may also suffer from social expectation bias, which leads respondents to choose the more socially acceptable answers. To alleviate this problem, we draw on the methods of Basuki et al. (2021) to detect the source of methodological bias by observing the most extreme response (MRS), which is the item with the highest factor load in the confirmatory factor analysis (Mishra, 2016). Four items are found to be MRS: Y1, X3, M11 and M22. When these items are excluded, the model parameters are recalculated, with the following results: Chi-square = 180.447, Df = 71, Chi-square/df = 2.54, CFI = 0.936, TLI = 0.917, SRMR = 0.042 and RMSEA = 0.066. These results are not significantly different from the previous ones. Hence, no social expectation deviation is believed to be present.

Furthermore, discriminative validity was considered acceptable when the AVE extracted by each construct (excluding shared values) was >0.5. The AVE of some indicators in this study was slightly lower than the discriminant validity threshold, but the CR values were all higher than 0.6, which could still be judged as acceptable value and validity (Lam, 2012).

## Findings

### Correlation analysis

Table 4 shows the correlation coefficients and variance inflation factor (VIF) values. EI was highly correlated with EE, ESE, EC, family income and whether parents had entrepreneurial experience (*p* < 0.01). ESE was closely related to EE, EC, family economic income and whether parents had entrepreneurial experience (*p* < 0.01). We also checked VIFs, all less than 3, showing no significant multicollinearity amongst the variables.

**Table 4:** Correlation coefficients and VIFs.

	1	2	3	4	5	6	7	8	9	10
Mean	0.48	2.18	2.90	3.79	3.89	0.28	0.45	5.1761	4.9444	4.2799
Std. dev	0.500	1.076	1.136	2.701	1.578	0.448	0.498	0.95612	1.10895	1.11571
VIF	1.112	1.188	1.072	1.079	1.057	1.094	1.139	1.629	1.984	1.659
1 Gender	1									
2 Income	−0.029	1								
3 Grade	0.018	0.187**	1							
4 Major	−0.214**	0.008	−0.007	1						
5 Hometown	0.118*	−0.085	−0.086	−0.001	1					
6 Onechild	0.005	0.228	0.002	0.336	0.024	1				
7 Parents	−0.059	0.294**	0.054	0.089	−0.052	0.068	1			
8 EE	−0.033	0.091	−0.005	−0.078	−0.020	−0.111*	0.089	1		
9 EC	−0.058	0.106*	0.061	−0.107*	0.052	−0.028	0.162**	0.594**	1	
10 ESE	0.035	0.241**	0.055	−0.050	0.016	0.005	0.180**	0.456**	0.583**	1
11 EI	−0.012	0.257**	0.033	−0.020	−0.042	−0.046	0.260**	0.444**	0.492**	0.689**

*N* = 351, **value of *p* < 0.01, *value of *p* < 0.05.

### Testing the hypotheses

We used Mediation Process Model 6 and Moderation Process Model 1, provided by Hayes (2018), to test the aforementioned hypotheses. SPSS process v3.5 was used. The confidence interval was set to 95%, and the number of iterations was 5,000. This method makes up for the shortcomings of the stepwise regression method and the Sobel test method as it does not require the assumption of normal distribution and has higher sensitivity. The test results are reported in Tables 5, 6.

**Table 5:** Bootstrapped moderated-mediation results.

			*β*	*p*	Hypothesis	(LLCI;ULCI)	R^2^
**Mediation**							
*H1*: EE(Total effect)	**→**	EI	0.6663	***	Supported	(0.5247, 0.8078)	0.1971
*H2*: EE	**→**	EC	0.6885	***	Supported	(0.5902, 0.7867)	0.3524
*H3*: EC	**→**	EI	0.0911	0.1805	Not Supported	(−0.424, 0.2246)	0.4989
*H4*: EE	**→**	ESE	0.1987	**	Supported	(0.0763, 0.3212)	0.3585
*H5*: ESE	**→**	EI	0.7549	***	Supported	(0.6349, 0.8749)	0.4989
*H6*: EC	**→**	ESE	0.4847	***	Supported	(0.3791, 0.5903)	0.3585
*H8*: EC + ESE(Direct effect)	**→**	EE **→** EI	0.2017	**	Supported	(0.0603, 0.3431)	0.4989
**Moderation**							
*H7a*: family income	**→**	EE **→** EI	0.1641	*	Supported	(0.0220, 0.3061)	0.2553
*H7b*: family income	**→**	ESE **→** EI	0.0953	*	Supported	(0.0082, 0.1824)	0.4907

***value of *p* < 0.001, ** value of *p* < 0.01, *value of *p* < 0.05. *β*, regression weight estimate; *p*, value of *p*; LLCI and ULCI, lower level and upper level of confidence interval; *R*^2^, multiple squared correlation indicating the percentage of variance explained.

**Table 6:** Measuring mediation effects.

	Indirect effect	(BootLLCI, BootULCI)	Conclusion	Proportion of mediating effect
EE → EC → ESE → EI	0.2519	(0.1781, 0.3374)	Partial mediating	37.81%
EE → ESE → EI	0.1500	(0.0538, 0.2564)	Partial mediating	22.51%
EE → EC → EI	0.0627	(−0.0301, 0.1674)	Not mediating	-

Without considering the mediation effect, EE was found to have a significant impact on EI (*β* = 0.6663, *p* < 0.001), thus supporting *H*1. The test results also showed that EE has a significant level of influence on ECs (*β* = 0.6885, *p* < 0.001); therefore, *H*2 was supported. Similarly, EE (*β* = 0.1987, *p* < 0.01) and EC (*β* = 0.4847, *p* < 0.001) both had positive effects on ESE, supporting *H*4 and *H*6. EC had no significant effect on EI (*β* = 0.0911, *p* > 0.1) but had a significant impact on ESE and EI (*β* = 0.7549, *p* < 0.001), thus rejecting *H*3 but supporting *H*5. Finally, under the influence of the mediating effect, although the direct effect of EE on EI was significant (*β* = 0.2017, *p* < 0.01), it was lower than the main effect, from 0.6663 to 0.2017, indicating that part of the mediating effect exists; therefore, *H*8 was supported.

In the moderating effect test, family income was significant in the relationship between EE and EI (*β* = 0.1641, *p* < 0.05) and that between ESE and EI (*β* = 0.0953, *p* < 0.001). To adjust the effect, it was assumed that *H*7a and *H*7b held.

### Further analysis of the mediation effect

Hypotheses *H*5 and *H*8 were supported, confirming that EC and ESE play a mediating role between EE and EI. Since *H*3 was rejected, the indirect effects of this model were only the chain indirect effect of EE → EC → ESE → EI and the indirect effect of EE → ESE → EI. Table 6 reports the specific indirect effect size and the proportion to the total effect. The proportion of the chain mediating effect (37.81%) was higher than that of univariate mediators (22.51%), and the sum of the two exceeded 60%, indicating that the mediation effect had a significant effect between EE and EI.

### Further analysis of the moderating effect

**Figure 2:**
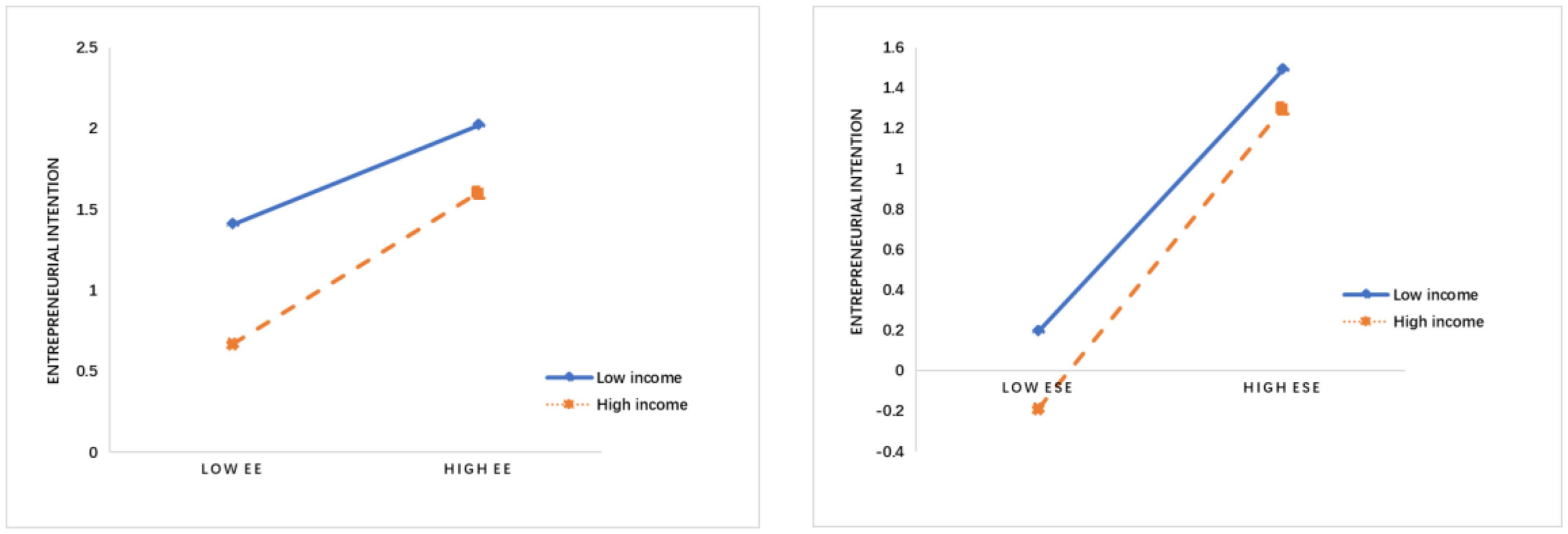
The moderating effect of family income in EE→EI and ESE→EI.

To further measure the moderating effect of family income, we used a group test method to measure the different effects of high income and low income on the effects of EE and ESE on EI. We divided family income as a categorical variable into two groups according to income and created a moderating effect diagram (Figure 2). The regression results showed that in the relationship between EE and EI, the regression coefficient of the ESE of the low-income group was 0.521, and the regression coefficient of the ESE of the high-income group was 0.868. In the relationship between ESE and EI, the regression coefficient of the ESE of the low-income group was 0.792, and the regression coefficient of the ESE of the high-income group was 0.961. The Chow test further supported these results, again validating hypotheses *H*7a and *H*7b.

## Discussion

We tested the seven hypotheses of this study by collecting questionnaire data from college students in China. We examined the mediating role of EC and ESE in the relationship between EE and EI and confirmed that ESE plays a significant mediating role between EE and EI. EE and EI are also affected through the chain mediating effect of EC and ESE. In addition, we confirmed that family income can positively moderate the relationship not only between EE and EI but also between ESE and EI.

### Theoretical implications

The results of this study show how EC and ESE, derived from EE, positively influence EI. The reasons behind this phenomenon are understandable, as subjective norms have a significant impact on both attitudes towards entrepreneurial behaviour and perceived control over that behaviour (Fernández-Pérez et al., 2017). Prior studies into engineering education (Barba-Sánchez and Atienza-Sahuquillo, 2018) and university students in developing countries (Memon et al., 2019; Mukhtar et al., 2021) came to similar conclusions as well.

Our research results emphasise the importance of ECs and ESE. ECs for college students are more similar to experiential entrepreneurial learning, and they provide knowledge and real experience that cannot be acquired in traditional classrooms. Therefore, these experiential projects have a significant positive effect on EI. By exploring how EC and ESE help EE to improve EI, we confirmed that the chain mediation effect of EC and ESE is higher than that of ESE.

In addition, we confirmed the moderating effect of family income on EI. Young people with high family income are more focused on their own careers, as they do not have to consider taking more risks (Wang et al., 2011), so family income can affect children’s entrepreneurial choices (Hsu et al., 2007). With the same degree of EE or ESE, students from well-off families were found to show increased EI (Figure 2), which not only confirms the moderating role of family income but also enriches the literature on EE and EI.

Finally, our research is valuable because we paid special attention to the regression effect of EC on EE, and the results showed a significant positive correlation (*β* = 0.6885***), proving that EC and ESE play a chain mediating role between EE and EI. This helps to explain why China encourages and promotes EC, and it also helps to explain the internal motivation of outstanding competition winners to become true entrepreneurs.

### Practical implications

Based on our findings, the effective implementation of EE can improve the EI of college students. The effective development of EE needs to start from two aspects. On the one hand, the development of EE requires a good entrepreneurial environment and a strong entrepreneurial atmosphere. Universities should strengthen cooperation with enterprises, further promote the integration of production and education and encourage college students to understand the latest technological developments and market demand to broaden their horizons and stimulate innovation and entrepreneurial thinking. This will work to enhance their internal motivation to engage in entrepreneurship. On the other hand, universities should attach importance to the construction of teacher teams who work to provide EE, regularly train and assess teachers engaged in EE, encourage teachers with ‘dual teachers and dual abilities’ qualifications to teach EE, hire entrepreneurs as entrepreneurship teaching consultants and ensure good quality EE.

In addition, we encourage EC in schools due to the stronger mediating effect of incorporating ECs into EE at the practical levels (37.81% >22.15%, Table 6). In ECs, EE can be well combined with practise. Students can hone their analytical abilities, professional abilities and judgement through ECs. Furthermore, teams with relatively low degrees of professionalism may be eliminated. Therefore, diversified cooperation is encouraged, and students can carry out diversified cooperation models across disciplines, institutions and regions to achieve professional complementarity and mutual promotion.

Our findings also showed that ESE plays a partial mediating role between EE and the establishment of EI (Table 6). Therefore, to improve the internal motivation of college students’ EI, the role of ESE cannot be ignored. We recommend that teachers and related personnel give students positive and valuable feedback in the classroom or in practise, improve the quality of teacher–student interactions, help students overcome difficulties in entrepreneurial learning and increase students’ participation in EC or other entrepreneurial drills or projects to enhance their confidence and ESE.

We also noted that family factors play a crucial role in the formation of college students’ EI. In our model, we verified that family income plays a moderating role in college students’ EI (Table 2). Therefore, we call for entrepreneurial policies to provide more support in the field of college students’ entrepreneurship to reduce the inhibitory effect of family factors on college students’ EI. Research has shown that entrepreneurial policies provide important support and guarantees in the cultivation of college students’ entrepreneurial ability (Lo and Tang, 2020). For example, universities can provide entrepreneurial loans (McKenzie and Sansone, 2017).

Finally, the EE of college students is multi-dimensional and requires the joint efforts of the government, society and schools to improve the EI of college students as a whole. This includes the synergising of policy support, teaching and EC support.

### Limitations and suggestions for future research

Despite the contributions of our study, it still has some limitations. The first is a limited threshold of viewpoints, as some factors may have been omitted from the selection of variables, which could have led to incomplete results. Second, due to the limitations associated with data collection, we assessed only college students in China. Therefore, it is difficult to generalise the research results to other environments and locales.

However, these limitations also create opportunities for future research. Future research can broaden our understanding of the factors affecting EE and EI and enrich the knowledge of the antecedents, mediators or moderators of EI. Second, it might be more interesting to study this phenomenon using qualitative methods. Finally, the same questionnaire can be administered to college students in other countries or regions to determine whether our findings differ from those in other countries or regions.

## Conclusion

This research empirically confirmed that the mechanisms promoting EI formation amongst Chinese college students are not only affected by EE but also by students’ ESE and EC. Although the influence of ECs on EI was not directly shown, they are related to ESE. The formed chain of mediation indirectly affects EI, and the effect of this chain of mediation is more significant than that of ESE alone. In addition, the formation of EI amongst Chinese college students is also affected by family income. This relationship complicates the formation of EI amongst college students. The model we constructed through theoretical derivation adequately explained the mechanisms impacting these factors.

Despite the exciting results of the study, there are still some limitations. In future research, researchers from other cultural backgrounds should conduct larger-scale surveys to bring additional value to the field. From this perspective, more researchers need to verify our results in the context of other cultures.

## Data availability statement

The raw data supporting the conclusions of this article will be made available by the authors, without undue reservation.

## Author contributions

YG completed most of the paper work. XQ was responsible for the collection of questionnaires. All authors contributed to the article and approved the submitted version.

## Funding

This research was funded by Guangdong Educational Science Planning Project (Higher Education Special), “Focusing on the cultivation of innovative talents in Guangdong, Hong Kong and Macao, creating a goal-oriented innovative teaching model” (grant number: 2021GXJK423).

## Conflict of interest

The authors declare that the research was conducted in the absence of any commercial or financial relationships that could be construed as a potential conflict of interest.

## Publisher’s note

All claims expressed in this article are solely those of the authors and do not necessarily represent those of their affiliated organizations, or those of the publisher, the editors and the reviewers. Any product that may be evaluated in this article, or claim that may be made by its manufacturer, is not guaranteed or endorsed by the publisher.
